# Effect of Size and Targeting Agent on Biodistribution of Polystyrene Nanoparticles in Apolipoprotein E Knock-Out and Wild-Type Mice

**DOI:** 10.3390/diagnostics15172140

**Published:** 2025-08-25

**Authors:** Harshvardhan Ajay Khare, Salime Bazban-Shotorbani, Tina Binderup, Andreas Kjaer, Nazila Kamaly

**Affiliations:** 1 Department of Clinical Physiology and Nuclear Medicine & Cluster for Molecular Imaging, Copenhagen University Hospital–Rigshospitalet & Department of Biomedical Sciences, University of Copenhagen, Blegdamsvej 3B, 2200 Copenhagen, Denmark; h.khare@sund.ku.dk (H.A.K.); tinbin@sund.ku.dk (T.B.); 2 Department of Health Technology, Danish Technical University, Ørsteds Plads, Building 345C, DK-2800 Kgs. Lyngby, Denmark; s.bazban-shotorbani19@imperial.ac.uk; 3 Department of Chemistry, Molecular Sciences Research Hub, Imperial College London, White City Campus, Wood Lane, London W12 0BZ, UK

**Keywords:** atherosclerosis, polystyrene nanoparticles, optical imaging, VCAM-1

## Abstract

**Objectives:** We investigated the in vivo biodistribution of vascular cell adhesion molecule-1 (VCAM-1)-targeting polystyrene nanoparticles (PS-NPs) labeled with Rhodamine B in a murine model of atherosclerosis. **Methods:** Targeted PS-NPs of varying sizes were first assessed for in vitro uptake in RAW264.7 cells. In vivo evaluation with VCAM-1-targeted nanoparticles (NP T) in C57 BL/6NtaC mice was conducted, and organs were analyzed 1, 6, and 24 h post injection, ex vivo. Subsequently, both targeted (NP T) and non-targeted (NP NT) nanoparticles of 30, 60, and 120 nm were injected into Apolipoprotein E knock-out (ApoE KO) mice on a high-fat diet, with ex vivo organ analysis 24 h post injection. **Results:** Results showed that NP30 T and NP60 T accumulated primarily in the liver and kidney of B6 mice. In ApoE KO mice, biodistribution was largely unaffected by size and targeting, except for higher uptake of NP 120 NT and T in the lungs and spleen. All NP types, except NP60 NT, showed significantly higher signal in ApoE KO mouse aortas compared to saline controls, with no significant differences between NP groups. **Conclusions:** While nanoparticles accumulated significantly in ApoE KO mouse aortas compared to controls, size and targeting properties did not significantly affect biodistribution in major organs 24 h post injection.

## 1. Introduction

Atherosclerosis remains to be the underlying cause of acute cardiovascular events [1]. It develops gradually over decades, driven by risk factors such as diet, smoking, hypercholesterolemia, and genetic predisposition [2,3,4]. The initiation of atherosclerotic lesions is believed to involve rolling, adhesion, and infiltration of immune cells with the help of adhesion molecules [5,6]. Amongst these, vascular cell adhesion molecule-1 (VCAM-1, CD106) is known to play a key role in the adhesion of monocytes and lymphocytes and is expressed under atherosclerotic conditions by activated endothelial cells and smooth muscle cells [7,8,9].

Targeting VCAM-1 for the development of non-invasive imaging techniques to identify atherosclerosis early in the process has been and continues to be a long-standing goal [10,11]. Several attempts have been made to develop and synthesize nanoparticles for various imaging modalities and therapeutic applications [6,10,12,13]. In vivo fluorescent imaging technologies offer a promising approach in the visualization of various atherosclerotic processes and targets at a cellular level without exposing the patient to radiation or invasive procedures, and they are relatively easier to work with [14]. Nanoparticles labeled with fluorescent dyes have the potential to achieve this goal given their plasticity, stealth properties, and a large surface area to carry a desired load [15,16]. Polystyrene nanoparticles (PS-NPs) have been widely used for their flexibility over modifications, a low polydispersity index, ease of synthesis, and commercial availability that make them advantageous for in vivo imaging studies [17]. Additionally, these nanoparticles display high colloidal stability at physiological conditions and a high drug loading capacity, making them suitable as nano drug delivery platforms. In a previous study, these nanoparticles were functionalized with three known VCAM-1-binding peptides, and the same nanoparticles were evaluated for their effect on binding and transport across in vitro models of endothelial dysfunction. It was observed that nanoparticles sized in the range of 30 to 60 nm showed enhanced permeability and binding, independent of peptide type or density [18].

In this study, we present our first in vitro and in vivo evaluation of PS-NPs for fluorescent imaging in murine models of atherosclerosis. Using intravenous administration of PS-NPs labeled with Rhodamine B (RhoB) in C57BL/6 (B6) mice and ApoE knockout (ApoE KO) mice, we investigate their biodistribution and assess the feasibility of using these nanoparticles for in vivo fluorescent imaging.

## 2. Materials and Methods

### 2.1. Nanoparticle Synthesis

Briefly, 500 μL of NP solution (1 wt%) polystyrene nanoparticles (PS-NPs) of three diameters (30 nm, 60 nm and 120 nm, micromod particle technology GmbH, Rostock, Germany) were washed twice in phosphate buffer (20 mM) with a pH of 8 with an Amicon ultra centrifugal filter unit (10 kDa cutoff, Millipore, MA, USA) at 10,000 RPM for 30 min at 4 °C and then dissolved in 200 μL of phosphate buffer. The nanoparticles were then PEGylated with a mixture of maleimide polyethylene glycol N-hydroxysuccinimidyl ester (Mal-PEG-NHS, 2 kDa, Nanocs Inc., New York, NY, USA) and methoxy polyethylene glycol N-hydroxysuccinimidyl ester (m-PEG-NHS, 2 kDa, Nanocs Inc.) (Mal-PEG-NHS, 2 kDa, Nanocs Inc.) with a molar ratio of 1:5 dissolved in 100 μL of dimethyl sulfoxide (Sigma-Aldrich, St. Louis, USA). The VCAM-1-targeting peptide (VHPKQHRGGGC with a molecular weight of 1175.34 Da) was coupled to the surface of functionalized NPs. Quantification of PEGylation, peptides on the surface of NPs, and measurement of size, zeta potential, and poly dispersity index were carried out in at least triplicates to meet quality requirements. The NP synthesis was based on the protocol previously used by Bazban-Shotorbani et al. [18].

### 2.2. In Vitro Uptake Study

For investigating the cellular uptake of NPs, RAW 264.7 cells were used. RAW 264.7 cells were grown on glass coverslips in a 12-well plate. They were incubated with RhoB-labeled nanoparticles (100 µg/mL) with a cell density of 1 × 10^5^ cells/mL for 24 h in an incubator at 37 °C at 5% CO_2_. Cells without RhoB-labeled NPs were grown in a different plate and considered as an experimental control. The suspension was removed after 24 h, and wells were washed three times using PBS buffer. The sample was fixed with 4% formaldehyde solution and washed three times. Diluted Hoechst (33342) in PBS buffer (1:5000, 1 mL/well) was added, and the cells were incubated for 15 min at 37 °C. The coverslips were fixed on the glass slides using ProLong Gold mounting medium, and a Zeiss LSM 710 confocal microscope (Carl Zeiss GmbH, Oberkochen, Germany) was used with a 63× oil immersion objective for imaging.

### 2.3. Ethical Statement for Animal Studies

All experiments involving animals were performed in compliance with the Animal Experiments Inspectorate in Denmark, Danish ministry of Justice (License no.: 2016-15-0201-00920), as well as in line with the regulations the ARRIVE guidelines, AVMA guidelines and regulations, and severity protocols.

### 2.4. Mouse Model of Atherosclerosis

Female ApoE KO mice (C57BL/6NTac background strain), 12 weeks old, were purchased from Taconic Biosciences (Germantown, New York, NY, USA) and allowed to acclimatize for one week, after which the diet was changed to a high-fat diet (D12492i, Research Diets Inc., NJ, USA) for 8 weeks. Female C57BL/6NTac (B6) mice, 7 weeks old, were purchased from Taconic Biosciences and were fed with normal chow diet. Simple randomization was used to allocate mice to different groups. All animal experiments were approved by the Danish animal authorities and in accordance with laws of the Danish Ministry of Justice for animal research as well as the ARRIVE guidelines.

### 2.5. Evaluation of NP Biodistribution in B6 Mice

**Figure 1:**
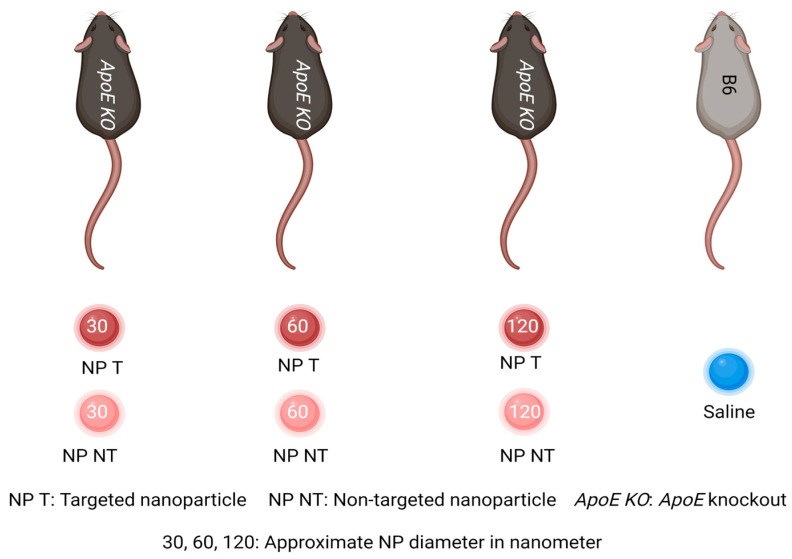
**Overview of in vivo study.** ApoE KO mice received both targeted nanoparticles (NP T) and non-targeted nanoparticles (NP NT) of all three sizes for the biodistribution study, where the B6 mice were injected with saline and were used as controls. NP 30T and NP60T were injected for the pharmacokinetic study in B6 mice only. 30, 60, and 120 are approximate nanoparticle diameters in nanometers (nm).

All the animals were anesthetized before the NP injections with a mixture of Midazolam (5 mg/mL, B. Braun Medical A/S, Frederiksberg, Denmark) and fentanyl (0.3 mg/mL, B. Braun Medical A/S, Frederiksberg, Denmark). B6 mice (*n* = 2 mice per group) were intravenously (IV, Tail) injected with 1 mg/mL of NP30 and NP60 nanoparticle formulations and allowed to circulate for 1 h and 24 h (for NP30) and for 6 h and 24 h (for NP60). The NP-injected animals were sacrificed post circulation period, and organs were immediately excised. Refer to Figure 1 for a basic overview of the in vivo study.

### 2.6. Biodistribution of NPs in ApoE KO Mice

All the animals were anesthetized before the NP injections with a mixture of Midazolam (5 mg/mL) and fentanyl (0.3 mg/mL). ApoE KO mice (*n* = 3/4 mice per group) were IV (Tail) injected with 1 mg/mL (Approximately 200 µL) of both targeted nanoparticles (NP30 T, NP60 T, NP120 T) and non-targeted nanoparticles (NP30 NT, NP60 NT, NP120 NT) and allowed to circulate for 24 h. The NP-injected animals were sacrificed post circulation period, and organs were immediately excised.

### 2.7. Ex Vivo Evaluation

The animals perfused with 10 mL saline through the left ventricle post sacrifice but pre-excision of whole organs. Whole organs from all the mice (liver, spleen, kidney, lung, heart, and aorta) were harvested for ex vivo imaging with an AmershamTM TyphoonTM Biomolecular Imager (GE HealthCare, Chicago, IL, USA) with a Cy3 560BP20 filter. Regions of Interest (ROIs) were drawn around the whole organ and analyzed as average or mean fluorescence intensity (MFI). Following ex vivo imaging, samples were further processed for histological analysis.

### 2.8. Immunofluorescence (IF) and Immunohistochemistry (IHC)

For paraffin-embedded tissue, organs were fixed in 4% formaldehyde solution immediately after ex vivo imaging for a maximum of 24 h. The paraffin-embedded tissue was sectioned with a thickness of 4 μm with a microtome. The tissue was then stained with DAPI at a concentration of 1:5000 with PBS. The sections were scanned on a Zeiss Axio Scan.Z1 (Carl Zeiss GmbH, Oberkochen, Germany) and scanned with a plan-apochromat 20× objective. Pre-programmed profiles for DAPI and Rhodamine B dyes were used with Zeiss ZEN Black software version 3.5.

### 2.9. Statistics and Data Analysis

Data are mean SEM unless otherwise stated. Non-parametric Mann–Whitney tests were carried out to study differences between two groups of mice. Statistical analysis was conducted using GraphPad Prism 9.0 software (GraphPad Software, Boston, MA, USA).

## 3. Results

### 3.1. In Vitro Uptake Study

**Figure 2:**
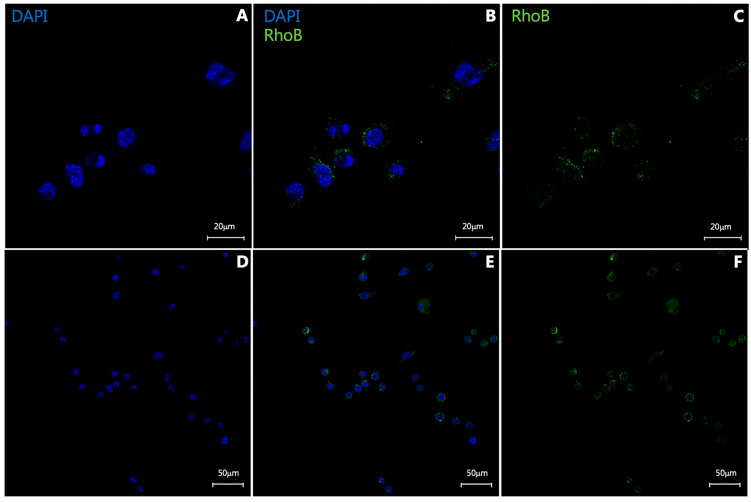
**In vitro uptake of RhoB-labeled NP60 T nanoparticles by RAW 264.7 macrophages.** DAPI (**A**,**D**) shows cells stained with nuclear stain only (in blue). Cells incubated with both DAPI and nanoparticles labeled with RhoB (in green, (**B**,**E**)). Cells incubated with RhoB-labeled nanoparticles only can be seen in (**C**,**E**). (**A**–**C**) are digitally zoomed images from lower panel (**D**–**F**) tile images (4 × 4 tile).

To assess whether PS-NPs are taken up by cells in vitro, the NP60Ts were incubated (100 µg/mL) with RAW 264.7 cells for 24 h at 37 °C. After the 24 h incubation, the cells were observed to have taken up the NPs (Figure 2B,E).

### 3.2. Biodistribution of PS-NPs in B6 Mice

**Figure 3:**
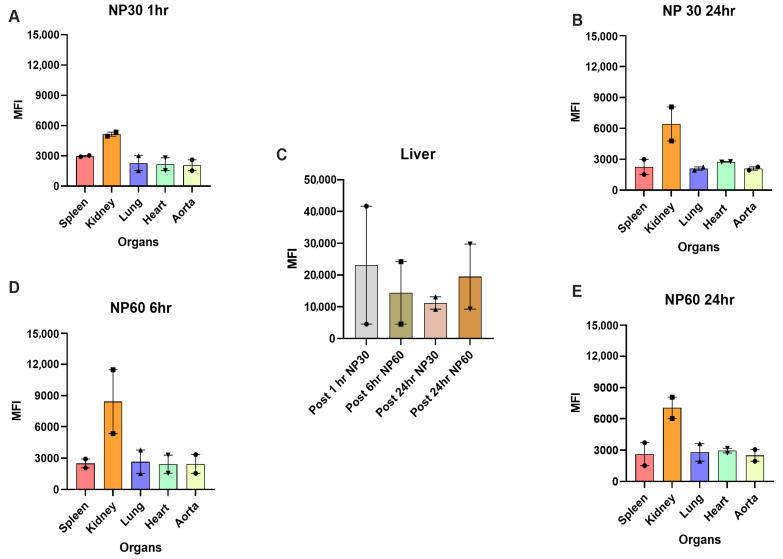
**Biodistribution of NP30 T and NP60 T in B6 mice.** NP30 T and NP60 T nanoparticles were considered for first in vivo injections to study biodistribution 1 h (NP30 T), 6 h (NP60 T), and 24 h (NP30 T and NP60 T) post injection in the liver, spleen, kidney, lung, heart, and aorta. (**A**,**B**) show NP30 T accumulation 1 h and 24 h post injection and Figure 2D,E show NP60 accumulation 6 h and 24 h post injection, respectively, where the kidney shows highest accumulation of NPs. (**C**) focuses on NP accumulation in the liver showing data on NP30 and NP60 accumulation 1 h, 6 h, and 24 h post injection. (**D**,**E**) show NP60 accumulation in various organs post 6 h and 24 h respectively. This study was performed in B6 mice (*n* = 2/group/time point). NP accumulation in terms of MFI (Median fluorescence intensity, data are mean SEM) values were observed.

Biodistribution analysis suggests that highest intensities were observed in the liver for all NP sizes at all studied time points (Figure 3C): NP30 T post 1 h (41,697 ± 9626), NP60 T post 6 h (24,209 ± 12,500), NP30 T post 24 h (13,126 ± 2310), and NP60 T post 24 (29,770 ± 546). These differences were found to be notable but non-significant. After the liver, the NPs accumulated in the kidney the most: NP30T after 1 h (5353 ± 41, Figure 3A), NP30 T after 24 h (8083 ± 1480. Figure 3B), NP60 T after 6 h (11,499 ± 6745, Figure 3D), and NP60 T after 24 h (Figure 3E). Accumulation in kidney possibly suggests blood filtration and elimination of NPs.

### 3.3. Tissue Biodistribution of NP30, NP60, and NP120 in Apoe KO Mice

**Figure 4:**
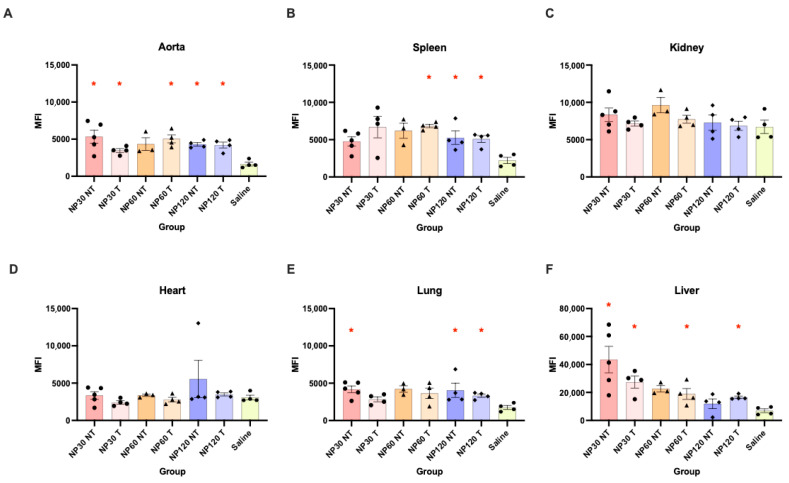
**Tissue biodistribution of targeted and non-targeted NP30, NP60, and NP120 in ApoE KO mice and saline-injected B6 mice.** (**A**) NP30 NT and NP60 T showed the highest accumulation in aortas. (**B**) Splenic accumulation of NP30T, NP120 T, and NP120 T was similarly lower compared to NP30 T, NP60 T, and NP60 T accumulation. (**C**) NP NT and NP T of all sizes seem to accumulate evenly in kidneys, with NP60 NT showing a slightly higher accumulation. (**D**) Similar to kidneys, NP accumulation in hearts is even across all sizes, types of nanoparticles and in both mouse types. (**E**) NP accumulation in the lungs is similar to the heart. (**F**) Highest accumulation of all nanoparticles observed in the liver. Stars over individual bars indicate significant differences compared to saline controls. Red asterisk indicates significant differences compared to saline-injected mice. N = 3/4 mice per group; data are presented in mean fluorescence intensity (MFI) values with standard error of mean.

**Figure 5:**
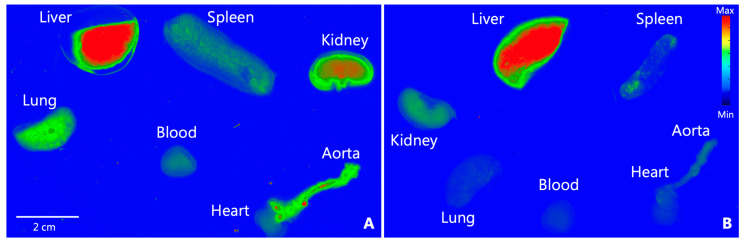
**Representative comparison between ex vivo whole organ fluorescent scans.** (**A**) Excised whole organs from a NP60 T-injected ApoE KO mouse compared to excised whole organs of an ApoE KO mouse injected with NP60 NT in (**B**), where similar accumulation of nanoparticles is observed in the liver of both the mice, but the kidney, spleen, lung, blood, and aorta show a higher signal in the NP60 T-injected ApoE KO mouse compared to the mouse injected with NP60 NT. The scans are in psudocolors, blue being the lowest median fluorescent intensity and red being the highest median fluorescent intensity. Scanned with AmershamTM TyphoonTM Biomolecular Imager (GE Healthcare, Boston, MA, USA).

All NPs except NP60 NT showed a significantly higher accumulation in the aortas of mice when compared to saline controls (Figure 4A), but there were no significant differences found when NPs of different sizes and targeting or non-targeting NPs were compared. Only NP60 T-, NP120 T-, and NP120 T-injected spleens showed significant differences compared to spleens of mice that were injected with saline (Figure 4B). No differences were found in kidneys and hearts across all NP types when compared to the same saline-injected organs nor between the NP groups (Figure 4C,D). NP120 T showed the highest accumulation in lung with NP30 NT, NP120 T showing a significantly higher accumulation compared to saline (Figure 4E). Liver was observed to be the organ of highest accumulation where biodistribution seemed to be affected the most by NP size (Figure 4F). NP30 NT displayed highest accumulation with NP30 T, NP60 T and NP120 T showing higher accumulation compared to saline-injected liver. A representative example of whole ex vivo organ scans shows accumulation of nanoparticles in various organs, and clear differences are observed compared to control organs, as observed in Figure 5A,B.

### 3.4. Histological Analysis of Tissue

**Figure 6:**
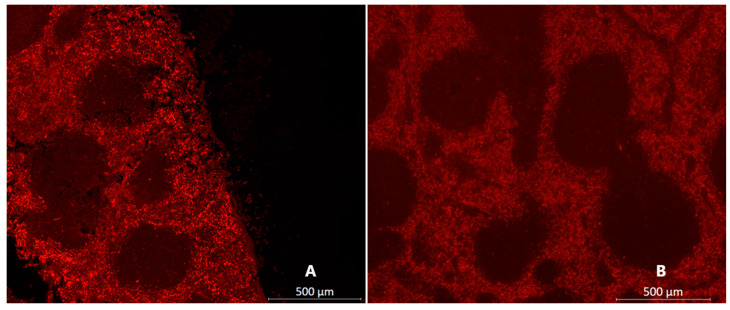
**Comparison between spleen of a mouse injected with NP versus spleen of a mouse injected with saline.** (**A**) Representative section of a NP60 T-injected spleen (NP signal in red) compared to a saline-injected spleen (in (**B**)). The sections were scanned on a Zeiss Axio Scan.Z1 and scanned with a plan-apochromat 20× objective. Pre-programmed profiles for DAPI and Rhodamine B dyes were used with Zeiss ZEN Black software version 3.5.

**Figure 7:**
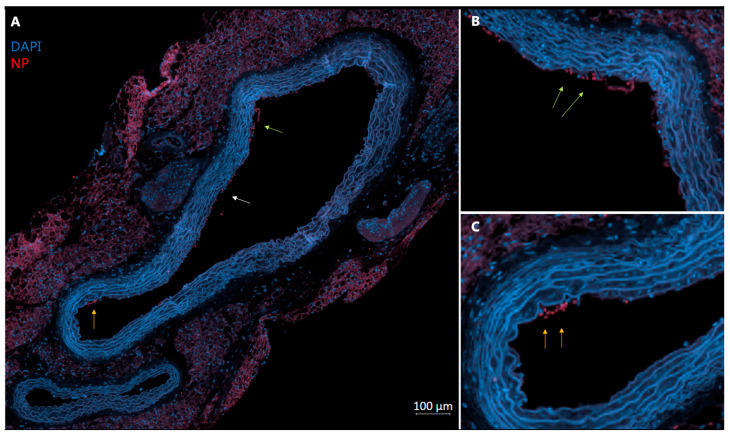
(**A**) **NP60 T in aorta.** Representative section of an aorta from a ApoE KO mouse injected with NP60 T (in red) and DAPI in blue. Arrows point to accumulation of NP60 T. (**B**,**C**) Magnified images from green and orange arrow showing sections of potential nanoparticle accumulation lining the endothelium. The sections were scanned on a Zeiss Axio Scan.Z1 and scanned with a plan-apochromat 20× objective. Pre-programmed profiles for DAPI and Rhodamine B dyes were used with Zeiss ZEN Black software version 3.5.

Fluorescent microscopic observations revealed presence of nanoparticles most prominently in the spleen (Figure 6) and liver. Aortic sections too revealed presence of nanoparticles along the inner lining of the vessel (Figure 7), albeit in less than liver and spleen. All the organs from saline-injected mice did not show the same signal intensity as shown by the organs of mice that were injected with NPs. The signal observed in saline-injected mice confirms the presence of autofluorescence.

## 4. Discussion

VCAM-1 is considered a promising target for non-invasive imaging of atherosclerosis since it is expressed by endothelial cells early in the atherosclerotic process and can therefore be targeted to change the course of disease [10,11,19]. In a previous in vitro study by Bazban-Shotorbani et al. involving VCAM-1-targeting PS-NP nanoparticles, NP30 T and NP60 T showed maximum binding of nanoparticles to a dysfunctional endothelium model based on HUVEC cells [18]. In the current study, in vitro investigation based on RAW264.7 cells demonstrated uptake of NP60 T nanoparticles, and the in vivo study concentrated on the biodistribution of PS-NPs of three sizes conjugated to VCAM-1-targeting peptide. This biodistribution study in B6 mice showed that the highest accumulation of NPs was in the liver across two sizes of NPs (NP30 T and NP60 T) 1 h, 6 h, and 24 h post injection. After the liver, the NPs accumulated preferentially in kidney across all time points of measurements post IV injections. Based on the biodistribution results in B6 mice, we decided to continue with biodistribution studies of NPs 24 h post IV injections. This study was conducted on ApoE KO mice that were relatively young (13 weeks old and fed with a high-fat diet for 8 weeks) to mimic a relatively early stage of atherosclerotic disease, where VCAM-1 expression seems to be upregulated [20,21]. In this atherosclerotic model, two versions (with a VCAM-1-targeting peptide, NP T, and a non-targeting nanoparticle, NP NT) of each size (30 nm, 60 nm and 120 nm) were evaluated. Differences in liver accumulation were found across all NP types. Otherwise, no significant differences were observed across all other organs and NP types. Ex vivo whole-organ imaging might not differentiate well between NP-injected organs compared to saline-injected organs, but microscopic analysis of tissue indeed suggests the presence of nanoparticles in spleen and liver compared to the same organs that were injected with saline. The results described in this study are consistent with the profiles of nanoparticles that have previously been described in the literature. Nanoparticles are typically cleared from the bloodstream and are taken up by residential macrophages, the spleen, and Kupffer cells of the liver [22,23,24,25]. It should also be noted that size and surface conjugation largely influence the in vivo characteristics of nanoparticles and their ability to evade the immune systems to reach the target. Nanoparticles with a size equal to or less than 100 nm are known to have favorable passive targeting due to the enhanced permeability and retention (EPR) effect [26,27]. Nanoparticle size is also known to affect the biodistribution properties in vivo, and this has not been observed to a great extent in our study. Apart from size, surface coating, too, affects the circulation and stability of nanoparticles in vivo. PEGylation has shown improvement in circulation time by protecting the nanoparticles from enzymes and antibodies to an extent, and we believe it has led to a longer circulation time and reduced liver and spleen uptake of nanoparticles [28,29]. Having said that, it might have also led to enhanced passive retention of nanoparticles. PEGylation has also shown to induce IgM responses, causing rapid and enhanced hepatic uptake of PEGylated nanoparticles. Therefore, there needs to be a perfect balance in PEGylation to achieve the perfect amount of stealthiness and circulation time of nanoparticles [30,31]. Previous attempts have been made to target VCAM-1 with nanoparticles for both diagnostic and therapeutic purposes, where VCAM-1-targeting nanoparticles delivered microRNAs to inflamed endothelial cells, confirmed by both in vitro and in vivo findings, where the addition of targeting peptides seems to alter the biodistribution of nanoparticles, resulting in non-specific uptake in the liver and kidneys [32,33,34].

There are a number of factors that are likely to influence the ability to detect these nanoparticles in vivo, and autofluorescence is a concern when imaging in the visible range, as observed in our study. Tissue autofluorescence has been observed in the saline-injected tissue in our study, which potentially affects the quantification of nanoparticle signal. Rho B labeling of NPs could have potentially influenced the detection of these NPs. Conjugation with RhoB resulted in a fluorescent signal in the 580 nm range that coincides with the autofluorescence emitted by blood and tissue components [35,36]. Additionally, there could also be potential differences in the background autofluorescence of the two strains, and these have not been calculated. The sensitivity of detection by the instrument, its potential inability to distinguish between two signals (nanoparticle versus autofluorescence), and the depth of penetration into the ex vivo tissue can all contribute to a loss of signal as well as the ability to measure the signal accurately. Near-infrared fluorescence imaging (NIRF) in the 700–900 nm range offers deep tissue penetration, up to several centimeters, and a considerable reduction in undesired tissue autofluorescence, which also results in an improved signal-to-background ratio [37,38]. The nanoparticles could be conjugated to NIRF labels for future studies. Imaging techniques such as PET coupled with nanoformulations provide higher sensitivity and tissue penetration, allowing for highly accurate non-invasive imaging of nanoparticles without having limitations of autofluorescence but also introduces radiation exposure. Labeling the nanoparticles with a radiolabel can be advantageous for biodistribution and targeting studies [39,40].

## 5. Conclusions

In the current study, we found that targeted and non-targeted PS-NPs in the size range of 30–120 nm accumulated in the aortas of ApoE KO mice and were significantly higher than saline controls.

Size and coating properties of the NPs were not found to affect the targeting or biodistribution properties 24 h post injection, and only a trend towards higher liver uptake of the smaller-sized NPs was observed. Due to autofluorescence and depth of penetration, Rhodamine B seems suboptimal for imaging atherosclerotic plaques, and near-infrared fluorophores might be preferred for future studies.

## Data Availability

The data presented in this study are available on request from the corresponding author.
